# Neutrophil percentage-to-albumin ratio is a potential marker of intravenous immunoglobulin resistance in Kawasaki disease

**DOI:** 10.1038/s41598-024-66135-5

**Published:** 2024-07-02

**Authors:** Linfan Deng, Ting Wang, Yan Duan, Bin Liu, Jun Jiang, Dong Liu, Gang Li

**Affiliations:** 1 Department of Pediatrics, The Affiliated Hospital of Southwest Medical University, No. 25 Taiping Street, Luzhou, Sichuan China; 2 Sichuan Clinical Research Center for Birth Defects, Luzhou, China; 3 Mianyang Central Hospital, School of Medicine, University of Electronic Science and Technology of China, Mianyang, Sichuan China; 4 Department of General Surgery (Thyroid Surgery), The Affiliated Hospital of Southwest Medical University, Sichuan, China; 5 Metabolic Vascular Diseases Key Laboratory of Sichuan Province, Luzhou, China

**Keywords:** Biomarkers, Cardiology, Risk factors

## Abstract

Intravenous immunoglobulin (IVIG) resistance in Kawasaki disease (KD) was associated with coronary artery lesions. Neutrophil percentage-to-albumin ratio (NPAR) is an index of mortality in several inflammatory diseases. This study focused on the association of NPAR with IVIG- resistance in KD. Clinical and laboratory data of 438 children with KD before IVIG treatment were retrospectively analyzed. Notably, high NPAR was associated with older age, high WBC, NP, ALT, total bilirubin and CRP, as well as with high the incidence of IVIG-resistance, and with low hemoglobin (Hb), PLT, ALB and sodium levels. NPAR (OR: 2.366, 95% CI: 1.46–3.897, p = 0.001) and Hb (OR: 0.967, 95% CI: 0.944–0.989, p = 0.004) were independent risk factors for IVIG-resistance. NPAR showed linear relation with IVIG-resistance (p for nonlinear = 0.711) and the nonlinear correlation was found between IVIG-resistance and Hb (p for nonlinear = 0.002). The predictive performance of NPAR was superior to Beijing model (z = 2.193, p = 0.028), and not inferior to Chongqing model (z = 0.983, p = 0.326) and the combination of NPAR and Hb (z = 1.912, p = 0.056). These findings revealed that NPAR is a reliable predictor of IVIG-resistance.

## Introduction

Kawasaki disease (KD) is characterized by systemic vasculitis and occurs predominantly in infants and children under 5 years old. KD is considered the most common cause of childhood-acquired heart disease in developed countries owing to its predisposition to coronary artery lesions (CALs). Although intravenous immunoglobulin (IVIG) can greatly decrease the incidence of CALs, more than 10% of patients with KD can develop IVIG-resistance^1^. It is well known that IVIG-resistance is more likely to lead to CALs; hence it is crucial to assess the risk of IVIG-resistance at an early stage so that aggressive treatment can be implemented to reduce the risk of CALs.

Although several serum inflammatory cytokines, including IL-6^2^, IL-41^3^, growth differentiation factor-15^4^ and tumor necrosis factor-α^5^, and systemic inflammatory parameters based on general laboratory data, such as neutrophil-to-lymphocyte ratio^1^, platelet-to-lymphocyte ratio^1^, prognostic nutritional index^6^ and C-reactive protein(CRP)/albumin (ALB) ratio^1^ have been attempted in many investigations to be used for the prediction of IVIG-resistance, these indicators are not perfect due to their low sensitivity and/or specificity. Therefore, the search for potential novel inflammatory predictors of IVIG-resistance is warranted.

Neutrophils increase under conditions of inflammation and infection and can induce the expression of various inflammatory mediators and cytokines. An increased neutrophil count is associated with IVIG-resistance^7,8^. Serum albumin levels are involved in antioxidant and anti-inflammatory effects, and low serum albumin levels were considered to be a predictor of IVIG-resistance^9^. Based on these findings, it is reasonable to speculate that the neutrophil percentage (NP)-to-albumin ratio (NPAR), a new biomarker of systemic inflammation, may be an ideal biomarker of IVIG-resistance in KD. Previous investigations have shown that NPAR could be used as an index of mortality in patients with cardiogenic shock^10^, acute kidney injury^11^, severe sepsis or septic shock^12^, atrial fibrillation^13^, stroke^14^, and myocardial infarction^15^. Additionally, Zhang et al. demonstrated that NPAR is an independent indicator of stroke-associated infections^16^. To our knowledge, no study has investigated the association between NPAR and IVIG-resistance, and it is unclear whether NPAR is superior to other Chinese risk scoring systems in predicting IVIG-resistance.

For all the above reasons, we hypothesized in the present study that (1) NPAR is associated with IVIG-resistance in children with KD and (2) NPAR is superior to other Chinese risk scoring systems for predicting IVIG-resistance.

## Materials and methods

### Patients

We retrospectively reviewed the medical records of 438 KD patients hospitalized at the Mian yang Central Hospital between January 2015 and June 2020. The diagnosis of complete and incomplete KD met the guidelines of the AHA 2004^17^. IVIG-resistance was indicated by the recurrence of fever ≥ 38 °C and recrudescence of one or more of the initial symptoms or persistent fever for > 36 h after completion of the initial IVIG treatment^17^. All patients received the same standard treatment of IVIG (2 g/kg) and oral aspirin (30–50 mg/kg) within ten days of illness onset, followed by low-dose aspirin (3–5 mg/kg) for 6–8 weeks if there was no CAL evidence. The exclusion criteria were as follows. The presence of other diseases, such as systemic autoimmune diseases, infectious diseases, metabolic and hematological diseases, tumors, kidney, other heart diseases, and malnutrition; II. glucocorticoid or other immunosuppressive drugs or IVIG treatment before admission; III. Incomplete laboratory data records during hospitalization. This study was approved by the Ethics Committee on Human Subjects at Mian Yang Central Hospital and was consistent with the 1975 Helsinki Declaration revised in 1996.

Laboratory data and baseline characteristics based on the latest test results before IVIG administration were collected. NPAR was defined as the ratio of NP to albumin. We calculated the Bei Jing model^18^ and Chong Qing model^19^. Bei Jing model (≥ 6 points) and Chong Qing model (≥ 2 points) were considered as higher risk scores for IVIG-resistance. Chong Qing model: PLT ≤ 278 × 10^9^/L (1 points), NP ≥ 80% (1 point), TB ≥ 18.5 μmol/L(1 point) and Na^+^ ≤ 135 mmol/L(1 point), if a total of ≥ 2 points were considered high risk for IVIG-resistance. Bei Jing model: CRP ≥ 90 mg/L (3 points), NP ≥ 70% (2.5 points), Na^+^  < 135 μmol/L (3 points), albumin < 35 g/L (2.5 points), and total bilirubin > 20 μmol/L (5 points), if a total of ≥ 6 points were considered high risk for IVIG-resistance. CALs were defined as Kobayashi Z-score ≥ 2.5 in the right coronary artery or left anterior descending coronary artery^20^.

### Statistical analysis

Statistical analyses were performed using SPSS version 22.0 (IBM Corp., Armonk, NY, USA), python software (version: scikit-learn 1.1.3) and R software (version 4.2.3). The data are expressed as the number (%) for categorical variables or as the median (25th–75th percentiles) for continuous variables. Categorical variables were compared with Chi-square test, while continuous data were analyzed using Mann–Whitney *U* test. Statistically significant parameters from univariate analysis were subjected to a multivariable model. We performed multivariate logistic regression in backward stepwise selection to identify independent risk factors for IVIG-resistance. The restricted cubic splines (RCS) was employed to assess the nonlinear relationships between independent risks and IVIG-resistance. Receiver operating characteristic (ROC) curve analysis based on python software (version: scikit-learn 1.1.3) was used to assess the predictive ability of the NPAR and other risk scoring systems. The discriminatory ability between the NPAR and other risk scoring systems was compared with that of the method of DeLong et al. Statistical significance was set at p < 0.05.

### Ethics statement

Written informed consent was obtained from all parents. This study was approved by the Ethics Committee on Human Subjects at the Mian Yang Central Hospital.

## Results

### Patient characteristics

Of the 438 patients with KD in this study, 40 cases were IVIG-resistant (9.1%). As shown in Table 1, compared with the IVIG-responsive group, the IVIG-resistant group had lower hemoglobin (Hb), ALB, and sodium levels, and higher NP and NPAR levels, as well as the incidence of CALs (All p < 0.05). There were insignificant differences between the two groups regarding gender, age, WBC, PLT, aspartate aminotransferase, Alanine aminotransferase (ALT), total bilirubin, CRP, ESR and the days of pre-IVIG fever, and the percentage of incomplete KD.

**Table 1:** Comparison of clinical data among the IVIG-responsive and IVIG-resistant groups in KD.

Variables	Total (n = 438)	IVIG-response (n = 398)	IVIG-resistance (n = 40)	*p*
Male, n (%)	271 (61.87)	249 (62.56)	22 (55.00)	0.348
Age (months)	24.00 (13.00, 42.00)	24.00 (13.00, 43.00)	29.00 (12.00, 37.00)	0.752
Days of pre-IVIG fever	6 (5–7)	6 (5–7)	6 (5–8)	0.083
WBC (10^9^/L)	13.92 (10.77, 17.26)	13.97 (10.95, 17.18)	13.35 (10.14, 17.77)	0.671
NP (%)	65.5 (52.19, 76.78)	65.26 (51.62, 76.06)	73.77 (60, 34, 82.56)	0.029
Hb (g/L)	105.00 (96.00, 113.00)	106.00 (97.00, 114.00)	96.00 (89.00, 103.00)	< 0.001
PLT (10^9^/L)	349.00 (267.00, 446.00)	358.00 (279.00, 444.00)	306.00 (216.00, 524.00)	0.26
AST (U/L)	33.00 (26.00, 51.00)	33.00 (26.00, 49.00)	43.00 (31.00, 53.00)	0.083
ALT (U/L)	27.00 (16.00, 70.00)	27.00 (16.00, 70.00)	27.00 (18.00, 68.00)	0.927
Total bilirubin (μmoL/L)	5.90 (4.00, 8.60)	5.90 (4.00, 8.60)	6.50 (4.700, 10.60)	0.186
ALB (g/L)	37.65 (33.89, 41.03)	37.65 (34.50, 41.07)	33.24 (29.28, 37.65)	< 0.001
CRP (mg/L)	74.71 (42.50, 128.32)	74.71 (41.03, 126.50)	97.60 (52.93, 151.78)	0.122
ESR (mm/h)	59.50 (46.00, 79.00)	59.50 (46.00, 78.00)	59.50 (45.00, 81.00)	0.531
Sodium (mmol/L)	136.90 (135.40, 138.20)	136.90 (135.70, 138.40)	136.00 (134.20, 136.90)	0.003
Incomplete KD, n (%)	83 (18.95)	75 (18.84)	8 (20.00)	0.859
CALs, n (%)	79 (18%)	68 (17.1%)	11 (27.5%)	< 0.001
NPAR	1.72 (1.33, 2.12)	1.70 (1.32, 2.09)	2.11 (1.62, 2.63)	< 0.001

*WBC* white blood cell, *NP* neutrophil percentage, *ESR* erythrocyte sedimentation rate, *CRP* C-reactive protein, *AST* aspartate aminotransferase, *ALT* alanine aminotransferase, *NPAR* neutrophil percentage-to-albumin ratio, *PLT* platelet counts, *Hb* hemoglobin, *ALB* albumin.

### Baseline characteristics of KD with low and high NPARs

All cases were divided into two groups based on the best cutoff value of NPAR in predicting IVIG-resistance. High NPAR was associated with older age, high WBC, NP, ALT, total bilirubin and CRP, as well as with high the incidence of IVIG-resistance and CALs, and with low Hb, PLT, ALB and sodium levels (All p < 0.05) (Table 2).

**Table 2:** Baseline characteristics of KD with low and high NPARs.

	Low NPAR (n = 389)	High NPAR (n = 49)	p
Male, n (%)	238 (61.18)	33 (67.35)	0.402
Incomplete KD, n (%)	77 (19.79)	6 (12.25)	0.204
Resistance, n (%)	27 (6.941)	13 (26.531)	< 0.001
CALs, n (%)	57 (14.7%)	22 (44.9%)	< 0.001
Days of pre-IVIG fever	6 (5–7)	6 (5–7)	0.06
Age (months)	22.00 (12.00, 39.00)	41.00 (23.00, 58.00)	< 0.001
WBC (10^9^/L)	13.81 (10.62, 17.03)	15.42 (11.14, 22.37)	0.038
NP (%)	63.19 (50.85, 73.41)	83.46 (79.47, 87.24)	< 0.001
Hb (g/L)	106.00 (96.00, 113.00)	100.00 (92.00, 110.00)	0.033
PLT (10^9^/L)	366.00 (287.00, 458.00)	265.00 (214.00, 365.00)	< 0.001
AST (U/L)	33.00 (26.00, 49.00)	39.00 (24.00, 57.00)	0.47
ALT (U/L)	27.00 (15.00, 64.00)	53.00 (22.00, 97.00)	< 0.001
Total bilirubin (μmoL/L)	5.90 (4.00, 8.30)	7.50 (4.700, 15.00)	0.002
ALB (g/L)	38.11 (35.39, 41.39)	29.40 (26.80, 31.79)	< 0.001
CRP (mg/L)	72.38 (39.70, 113.45)	142.44 (75.90, 187.50)	< 0.001
ESR (mm/h)	59.50 (46.00, 78.00)	66.00 (44.00, 82.00)	0.555
Sodium (mmol/L)	136.90 (135.80, 138.30)	135.80 (133.10, 137.20)	0.002

*WBC* white blood cell, *NP* neutrophil percentage, *ESR* erythrocyte sedimentation rate, *CRP* C-reactive protein, *AST* aspartate aminotransferase, *ALT* alanine aminotransferase, *NPAR* neutrophil percentage-to-albumin ratio, *PLT* platelet counts, *Hb* hemoglobin, *ALB* albumin.

### Multivariable logistic regression for predicting IVIG-resistance

**Figure 1:**
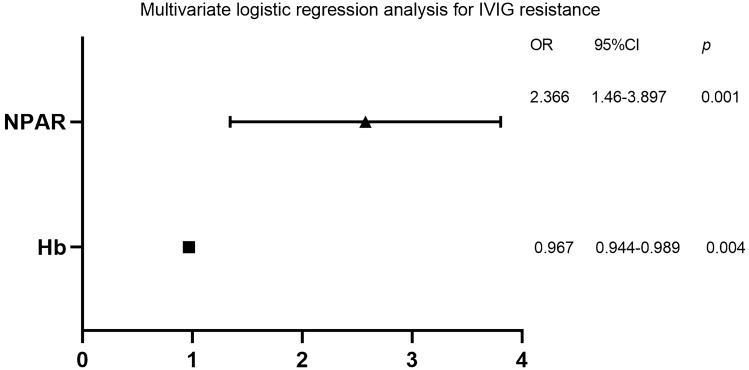
Multivariate logistic regression analysis for IVIG-resistance.

Variables with a p < 0.05 in univariate analysis were subjected to a multivariable model (Since ALB and NP have been included in NPAR, they were excluded from the multivariable analysis). Multivariable logistic regression showed that NPAR (OR: 2.366, 95% CI: 1.46–3.897, p = 0.001) and Hb (OR: 0.967, 95% CI: 0.944–0.989, p = 0.004) were independent risk factors for IVIG-resistance (Fig. 1).

### Nonlinear analysis

**Figure 2:**
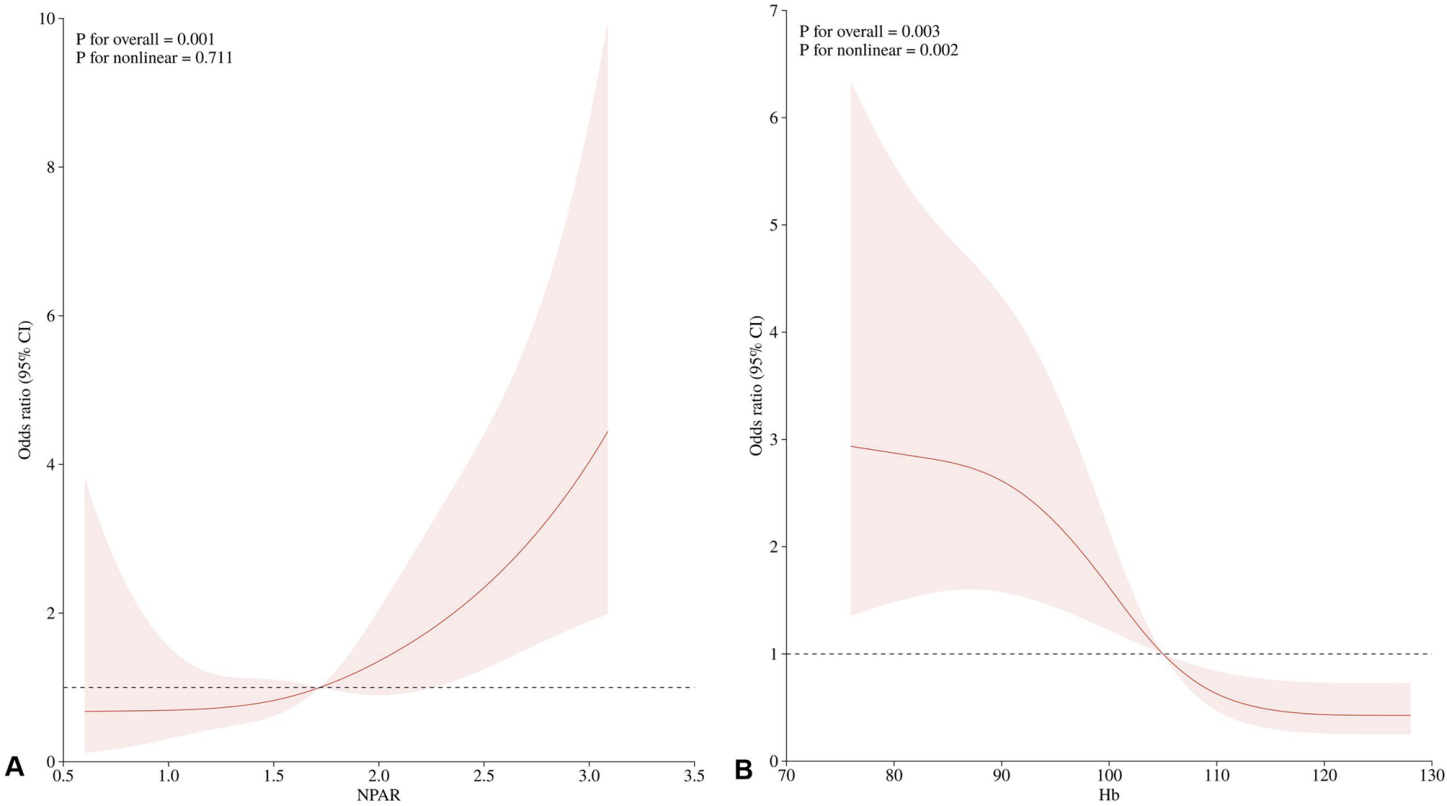
Nonlinear associations between independent risk factors and IVIG-resistance.

We found a clear dose–response relationship between Hb and IVIG-resistance (Fig. 2B). However, there was a linear association between NPAR and IVIG-resistance (Fig. 2A).

### The predictive power of NPAR, Bei Jing model and Chong Qing model

**Figure 3:**
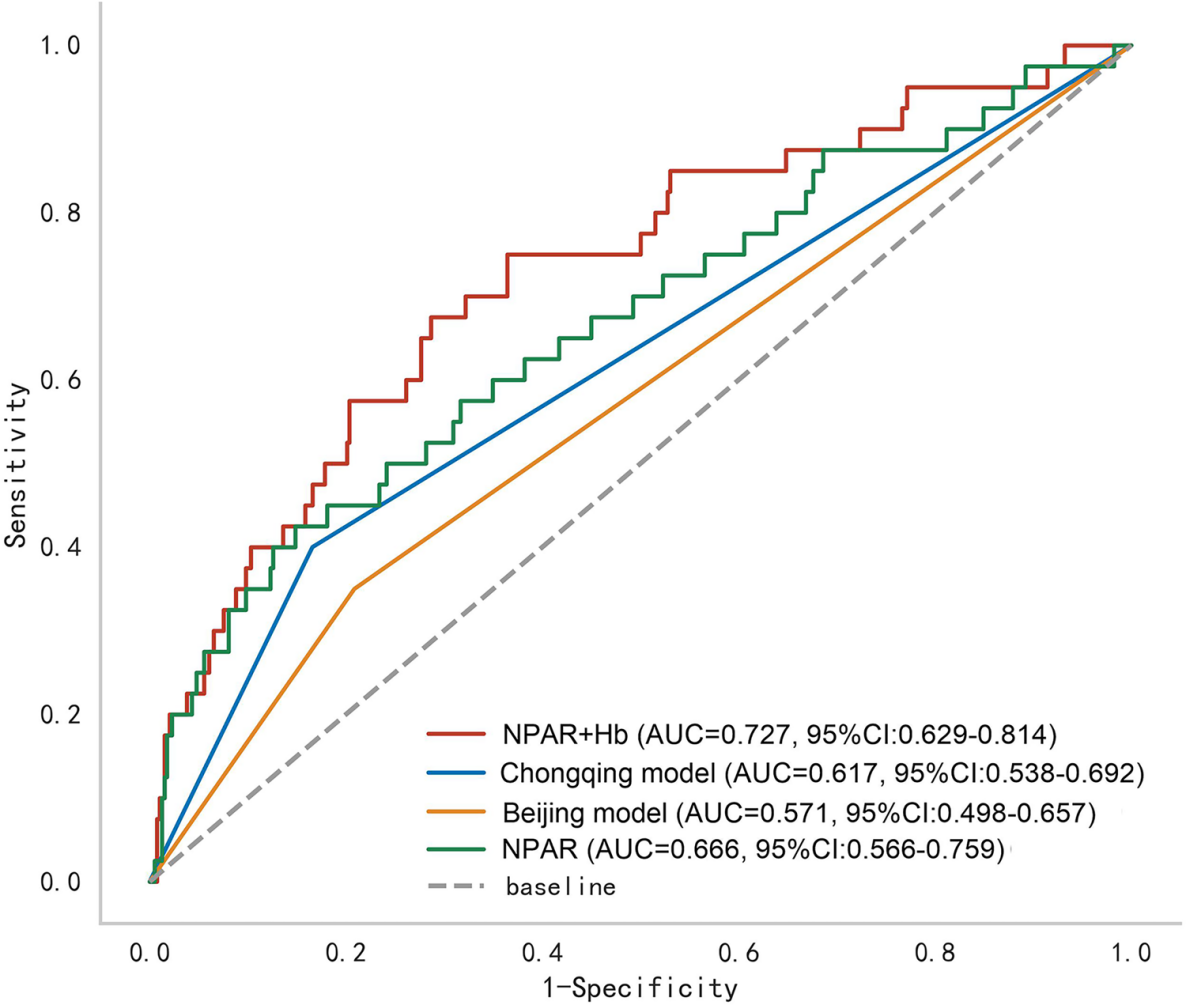
Receiver operator characteristic curves of NPAR, NPAR plus Hb, Bei Jing model and Chong Qing model for IVIG-resistance.

The area under the curve (AUC) value for NPAR to predict IVIG-resistance was 0.666, yielding a sensitivity of 42.5% and a specificity of 85.2%. NPAR displayed the highest sensitivity and specificity compared to Bei Jing model and Chong Qing model. The AUC of NPAR was superior to Beijing model (z = 2.193, p = 0.028), and not inferior to Chongqing model (z = 0.983, p = 0.326) and the combination of NPAR and Hb (z = 1.912, p = 0.056) to predict IVIG-resistance (Table 3 and Fig. 3).

**Table 3:** Values of NPAR and other prediction modes on the basis of ROC curves.

	AUC	Sensitivity (%)	Specificity (%)
NPAR	0.666	42.5	85.2
NPAR + Hb	0.727	67.5	71.4
Chongqing model	0.617	40	83.4
Beijing model	0.571	35	79.1

*NPAR* neutrophil percentage-to-albumin ratio.

## Discussion

To our knowledge, this retrospective study is the first to indicate that NPAR is independently associated with IVIG-resistance in children with KD. Additionally, NPAR was superior to the Beijing model and not inferior to the Chongqing model to predict IVIG-resistance.

Previous studies investigation revealed that 10–20% of patients with KD develop IVIG resistance after initial IVIG treatment^1^, which was close to our study. The association of neutrophils with KD was found^21,22^ and an increased risk of IVIG resistance among KD patients is associated with higher neutrophil counts^7,8,23^. Similarly, the IVIG-resistant group in the present study had a higher neutrophil percentage than the IVIG-responsive group. Albumin is a known nutritional status parameter. However, an evident association between albumin levels and inflammation was discovered in a recent study^24^. Hypoalbuminemia is prone to injure the immune function, thereby increasing the risk and development of infection. Low albumin levels are also associated with IVIG resistance^9,25^. Consistent with these findings, our results showed that the IVIG-resistant group had lower albumin levels than the IVIG-responsive group. Here are some reasons to explain this result. Albumin is inversely associated with the severity of acute inflammation^26^. Vascular leakage is considered a significant characteristic of KD pathophysiology, resulting in decreased albumin levels^26^. Furthermore, levels of vascular endothelial growth factor (VEGF) may serve as an indicator of the extent of vascular leakage. Elevated serum VEGF concentrations in KD patients have been correlated with reduced serum albumin levels^27^.

Based on this evidence, we explored the utility of NPAR integrating two inflammatory markers (i.e., neutrophil percentage and albumin) to predict IVIG-resistance. NPAR is a new inflammation-based prognostic score that accurately reflects the level of inflammation. Several studies have demonstrated the prognostic ability of NPAR in acute kidney injury^11^, liver cirrhosis^28^, cardiogenic shock^10^, and myocardial infarction^15^. Similarly, our study showed that NPAR was significantly elevated in IVIG-resistant patients. A high NPAR was associated with an increased risk of IVIG-resistance and high inflammatory markers (WBC, NP and CRP). In addition, low ALB and sodium were predictors of IVIG-resistance and associated with high NPAR in this study. More importantly, NPAR was an independent biomarker for IVIG-resistance, with an AUC of 0.666, a sensitivity of 42.5%, and a specificity of 85.2% for predicting IVIG-resistance in the present study, and was linearly associated with the IVIG-resistance, suggesting that NPAR can predict IVIG-resistance. In addition, our findings revealed that low hemoglobin was non-linearly associated with a high risk of developing IVIG-resistance, a result that may have occurred because heparin can induce inflammatory anemia^29^, and increased plasma heparin has been found in IVIG-resistant patients^30^.

The utility of predictive models to predict IVIG-resistance lies in their ability to identify patients who are expected to benefit from additional initial treatment regimens^31^. The Egami, Sano and Kobayashi risk scores based on usual laboratory data are widely used to predict IVIG-resistance in Japan, and their performance in non-Japanese populations is unsatisfactory due to ethnic and other differences^32^. Therefore, clinicians are tasked with identifying a dependable predictive model or biomarker for IVIG-resistance that is applicable to the specific region. In the present study, we compared the NPAR with two Chinese models (Bei Jing model and Chong Qing model) established by relatively large sample in two regions in China. It was noteworthy that NPAR was superior to Beijing model, and not inferior to Chongqing model and the combination of NPAR and Hb to predict IVIG-resistance. In contrast, NPAR had has fewer indicators. NPAR can be easily calculated from routine laboratory tests and does not add additional time and cost. Therefore, NPAR may be a more easily accessible and reliable biomarker of IVIG resistance in the Mianyang region in clinical practice.

This study had several limitations. First, this was a single-center retrospective study in Chinese population, and selection bias was inevitable. Second, the sample size was relatively small, which may have affected statistical validity. Therefore, prospective multicenter studies with larger sample sizes are required to validate these findings. Third, although we used multivariate modeling to control bias, many other known and unknown confounders may still exist. Fourth, neutrophil percentage and albumin usually remain dynamic before IVIG administration; therefore, random errors might be unavoidable.

## Conclusions

Our study revealed that NPAR was a reliable predictor of IVIG-resistance in KD.

## Data Availability

The datasets generated and/or analyzed during the current study are available from the corresponding author on reasonable request.

## Code availability

The software used in this study: https://www.xsmartanalysis.com/.
